# Mitochondrial Membrane Potential in Human Neutrophils Is Maintained by Complex III Activity in the Absence of Supercomplex Organisation

**DOI:** 10.1371/journal.pone.0002013

**Published:** 2008-04-23

**Authors:** Bram J. van Raam, Wim Sluiter, Elly de Wit, Dirk Roos, Arthur J. Verhoeven, Taco W. Kuijpers

**Affiliations:** 1 Department of Blood Cell Research, Sanquin Research and Landsteiner Laboratory, Amsterdam, The Netherlands; 2 Emma Children's Hospital, Academic Medical Centre, Department of Paediatric Immunology, University of Amsterdam, Amsterdam, The Netherlands; 3 Department of Biochemistry, Mitochondrial Research Unit, Erasmus MC, Rotterdam, The Netherlands; Massachusetts General Hospital and Harvard Medical School, United States of America

## Abstract

**Background:**

Neutrophils depend mainly on glycolysis for their energy provision. Their mitochondria maintain a membrane potential (Δψ_m_), which is usually generated by the respiratory chain complexes. We investigated the source of Δψ_m_ in neutrophils, as compared to peripheral blood mononuclear leukocytes and HL-60 cells, and whether neutrophils can still utilise this Δψ_m_ for the generation of ATP.

**Methods and Principal Findings:**

Individual activity of the oxidative phosphorylation complexes was significantly reduced in neutrophils, except for complex II and V, but Δψ_m_ was still decreased by inhibition of complex III, confirming the role of the respiratory chain in maintaining Δψ_m_. Complex V did not maintain Δψ_m_ by consumption of ATP, as has previously been suggested for eosinophils. We show that complex III in neutrophil mitochondria can receive electrons from glycolysis via the glycerol-3-phosphate shuttle. Furthermore, respiratory supercomplexes, which contribute to efficient coupling of the respiratory chain to ATP synthesis, were lacking in neutrophil mitochondria. When HL-60 cells were differentiated to neutrophil-like cells, they lost mitochondrial supercomplex organisation while gaining increased aerobic glycolysis, just like neutrophils.

**Conclusions:**

We show that neutrophils can maintain Δψ_m_ via the glycerol-3-phosphate shuttle, whereby their mitochondria play an important role in the regulation of aerobic glycolysis, rather than producing energy themselves. This peculiar mitochondrial phenotype is acquired during differentiation from myeloid precursors.

## Introduction

Neutrophils are the main phagocytic cells of the human immune system. These cells are the first to arrive at a site of infection, where they kill ingested microorganisms by producing superoxide through a rapid, NADPH (nicotinamide adenine dinucleotide phosphate, reduced) oxidase-mediated, respiratory burst, in combination with an arsenal of proteolytic enzymes and anti-microbial peptides [1], [2]. Neutrophils derive most of the energy required for these and other functions from a high rate of glycolysis [3]. This ensures that neutrophils can function in an inflammatory environment where the oxygen tension may be low or even absent [3]–[5].

Neutrophils contain relatively few mitochondria, which form a complex network inside the cell [6], [7]. It has been assumed that these organelles do not produce energy by respiration, as they do in other cells [3], [6]. However, neutrophil mitochondria can be stained with various mitochondria-specific dyes and do maintain a transmembrane potential (Δψ_m_), which is normally associated with respiratory chain activity and oxidative phosphorylation (OXPHOS) of adenosine diphosphate (ADP) [6], [7].

Δψ_m_ is generated by a series of four protein complexes (complex I-IV, respectively) in the mitochondrial inner membrane, which transfer electrons derived from the oxidation of reduced nicotinamide adenine dinucleotide (NADH) by complex I (NADH-ubiquinone oxidoreductase) or from the oxidation of succinate by complex II (succinate-ubiquinone oxidoreductase). Complex I, complex III (ubiquinol-cytochrome *c* oxidoreductase) and complex IV (cytochrome *c* oxidase) are the proton exchangers that maintain Δψ_m_ by transferring electrons to a subsequent complex or to molecular oxygen, with a concomitant transfer of protons out of the mitochondrial matrix. The final complex of the OXPHOS machinery, complex V (F_1_/F_0_-ATPase), is a very efficient molecular motor that allows the protons to flow back through its subunit F_0_ while utilizing the proton gradient for the phosphorylation of ADP to adenosine triphosphate (ATP) in the F_1_ subunit [8], [9].

Δψ_m_ can also be maintained by the adenosine nucleotide transporter (ANT) in collaboration with complex V. ANT is responsible for transporting ADP into the mitochondrial matrix in exchange for ATP. However, in the absence of respiration, ANT can transport ATP^4−^ into the matrix in exchange for ADP^3−^ and thus maintain Δψ_m_ by a net influx of negative charges [9]–[12]. This mechanism is only functional if the F_1_-ATPase provides the necessary ADP by hydrolysing ATP, but it is independent of the proton exchanger F_0_.

Recent evidence has indicated that respiratory chain complexes can be organized in larger supercomplexes in the mitochondrial inner membrane [13]–[17]. Supercomplexes of different composition have been described in various organisms, including yeast and mammals [18], [19]. In bovine heart and human skeletal muscle, these supercomplexes appear to be composed of a complex I monomer, a complex III dimer and up to four copies of complex IV, representing the largest forms of OXPHOS complex assemblies, also termed ‘respirasomes’ [14], [15], [17], [20]. The inclusion of complex IV in the supercomplex significantly enhances the activity of complex I and complex III [13], [14]. Thus, the efficiency of electron transfer along the respiratory chain is largely dependent on the composition of these respiratory supercomplexes.

Resting neutrophils consume little or no oxygen, and classical inhibitors of respiration, such as cyanide (KCN), an inhibitor of complex IV, do not affect neutrophil functions [3], [6]. In addition, neutrophil mitochondria contain very little cytochrome *c*, which would indicate a very low complex IV content and inefficient respiration [6], [21]. However, prolonged incubation with oligomycin (longer than 2 hours), an inhibitor of the F_0_-ATPase of the respiratory chain, does affect both NADPH oxidase activity and neutrophil chemotaxis [7]. This suggests that complex V is active and may play an important role in maintaining neutrophil functions.

In this report, we investigated how neutrophils maintain Δψ_m_ and whether their mitochondria still play a role in the regulation of their energy metabolism. We show that Δψ_m_ in neutrophils is maintained by residual respiratory chain activity, even though this activity is apparently hardly coupled to ADP phosphorylation. In addition, we show that the respiratory chain complexes in neutrophils lack supercomplex organisation, while these supercomplexes are present in peripheral blood mononuclear cells (PBMC) and the myeloid cell line HL-60. During differentiation to neutrophil-like cells, HL-60 cells loose mitochondrial supercomplex organisation, while gaining neutrophil-like abilities. We suggest that the lack of mitochondrial supercomplex organisation and the consequent lack of respiration is a requirement to maintain a high rate of aerobic glycolysis in order to fulfil the primary human neutrophil functions.

## Results

### Neutrophils retain some respiratory chain complex activity

**Figure 1:**
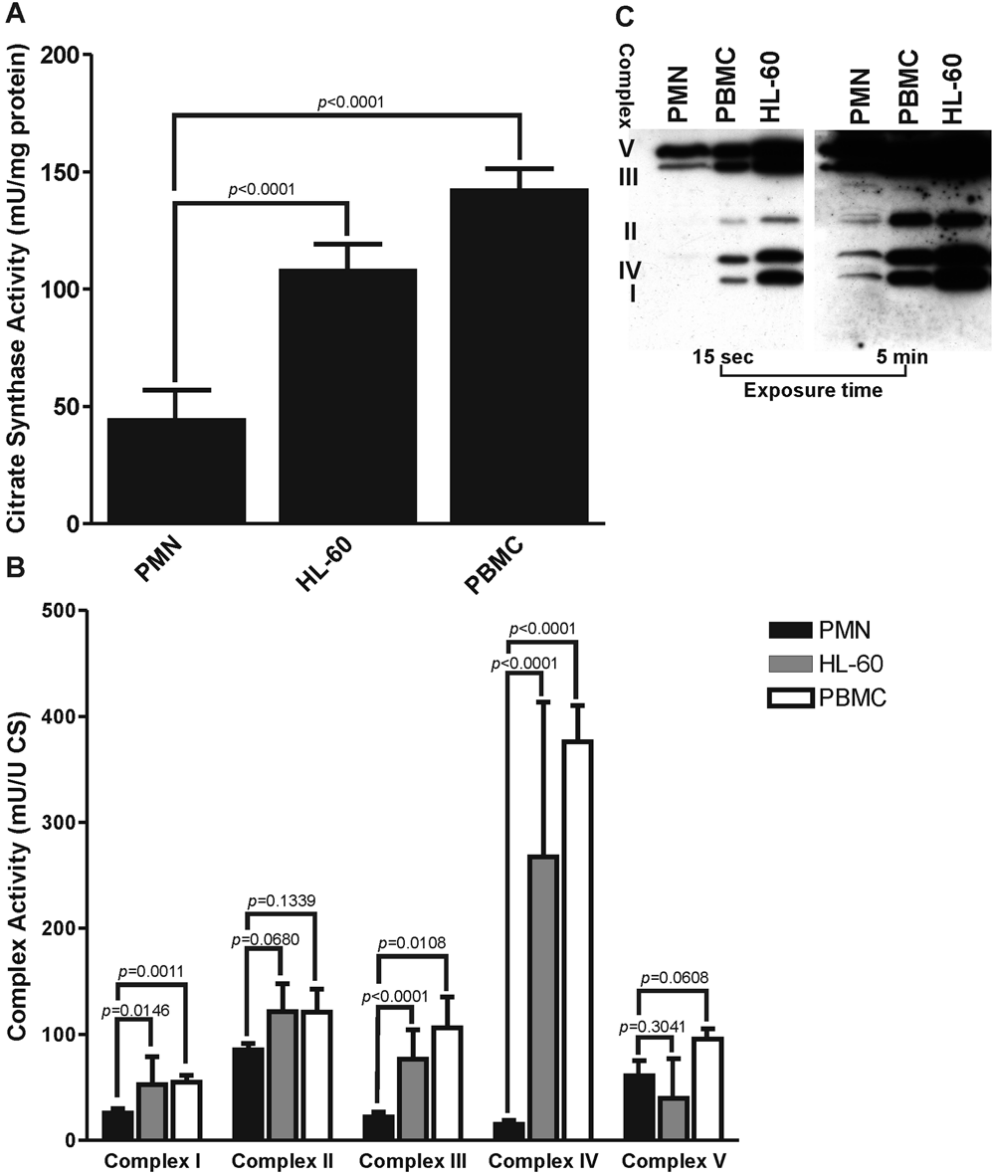
Mitochondrial content and respiratory chain enzyme activity in neutrophils, PBMC and HL-60 cells. A) Neutrophils (PMN) contain significantly less mitochondria as expressed per unit citrate synthase activity. Data represent the mean (±SD) of 13 (PMN), 6 (HL-60) or 16 (PBMC) different experiments performed in duplicate. B) Neutrophils retain full complex II and V activity while the activity of the remaining complexes is significantly reduced. Data represent the mean (±SD) of 10 different experiments performed in duplicate. Values were corrected for mitochondrial protein content and citrate synthase activity. C) Crucial subunits of the respiratory chain can be detected in neutrophil lysates on Western blot. 20 µg of mitochondrial protein was separated by SDS-PAGE and immuno-detected with anti-complex I (20 kDa), complex II (30 kDa), complex III (Core 2, 47 kDa), complex IV (COX II, 26 kDa) and complex V (α, 55 kDa) subunit antibodies. Blots exposed for 15 sec and 5 minutes are representative of three independent experiments.

To establish whether the respiratory chain could be responsible for maintaining Δψ_m_ in neutrophils, we determined the activity of the OXPHOS complexes in the isolated mitochondria of neutrophils, PBMC, and HL-60 cells. Although the mitochondrial content, as expressed by citrate synthase activity, was significantly reduced in neutrophils as compared to the other cells (Figure 1A), neutrophils still displayed respiratory chain enzyme activity. Complex II and complex V (F_1_) displayed normal levels of activity, while some complex I and complex III activity was detected as well (Figure 1B). The activity of complex IV was severely reduced in neutrophils as compared to the control cells. These results are in line with the observation that neutrophil mitochondria contain very little cytochrome *c*, an essential component of complex IV [6], [21].

Even though complex I, III and IV displayed very little enzymatic activity, Western blotting showed the presence of the cornerstone subunits of these complexes in neutrophil mitochondria lysates, although the total amount of the OXPHOS proteins that could be detected on blot was significantly less than in the control cells (Figure 1C).

### Respiratory chain complexes in neutrophil mitochondria are not organised in supercomplexes

To visualise the organisation of the respiratory chain complexes in neutrophils and the control cells, isolated mitochondria from these cells were solubilised with digitonin before subjecting the samples to blue native(BN)-PAGE. The BN-PAGE gels were subsequently processed by denaturating SDS-PAGE to resolve the (super)complexes into their respective subunits, before visualising the essential subunit of each of the five OXPHOS complexes by immunoblotting.

**Figure 2:**
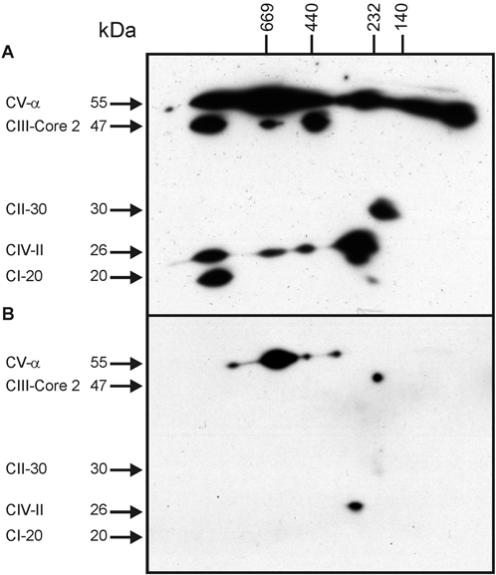
Supercomplex organisation of OXPHOS complexes in PBMC and neutrophils. A–B) Mitochondria isolated from PBMC (A) and neutrophils (B) were solubilized in digitonin, and 200 µg of mitochondrial protein was separated by BN-PAGE. Strips from the first dimension were excised and used for second dimension SDS-(12%)PAGE, transferred to PVDF membrane and immuno-detected with anti-complex I (20 kDa), complex II (30 kDa), complex III (Core 2, 47 kDa), complex IV (COX II, 26 kDa) and complex V (α, 55 kDa) subunit antibodies. Blots are representative of three independent experiments. High molecular weight purified proteins were used as molecular mass markers in the first dimension BN-PAGE: thyroglobulin, 669 kDa; ferritin monomer, 440 kDa; catalase 232 kDa; lactate dehydrogenase, 140 kDa.

In PBMC, complex III appears to form three distinct supercomplexes with complex IV of ∼1.5 MDa, ∼600 kDa and ∼470 kDa (Figure 2A). Fully assembled complex I is only present in the largest of these three supercomplexes (∼1.5 MDa), but a subcomplex of about 370 kDa of complex I in association with complex IV is also demonstrable. Complex II is not present in any supercomplex with the other respiratory chain complexes, as expected. Complex V showed apparent molecular masses of ∼1.5 MDa (dimer), ∼600 kDa (monomer), 470 and 370 kDa (F_1_ subcomplexes), and even less (Figure 2A).

Complex III and complex IV appear outside any supercomplex in neutrophils, having apparent molecular masses of ∼230 and ∼370 kDa, respectively (Figure 2B). The level of expression of complex I and II was below the detection limit on the neutrophil blot (Figure 2B). In neutrophils, complex V was present in four super- and subcomplexes with molecular masses ranging from 1.5 MDa to 370 kDa, but the level of expression of these complexes was significantly less than in PBMC.

### Activity of complex III but not V is required for maintaining Δψ_m_ in neutrophil mitochondria

The fluorescent dye JC-1 (5,5′,6,6′-tetrachloro-1,1′,3,3′-tetraethyl-imidacarbocyanine iodide) was used to determine Δψ_m_ in freshly prepared cells. JC-1 forms red fluorescent J-aggregates when the positively charged dye accumulates at high concentration in functional mitochondria, while the monomeric form is green fluorescent at lower concentrations. Thus, changes in the ratio between the intensity of the red (Fl-2) to green (Fl-1) fluorescence indicate changes in Δψ_m_.

**Figure 3:**
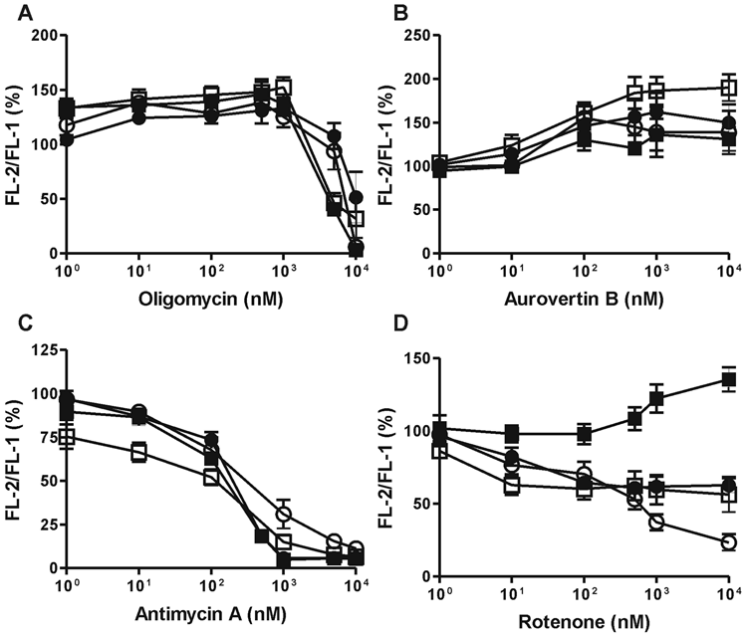
Mitochondrial membrane potential in neutrophils and PBMC. A–D) Neutrophils (▪,□) and PBMC (•,○) were incubated with (▪, •) or without (□, ○) 5 mM glucose. After 2 hours of pre-incubation at 37°C, the cells were incubated for an additional 2 hours in the presence of various concentrations of respiratory chain enzyme inhibitors. Afterwards, the cells were stained with the fluorescent dye JC-1, and fluorescence in Fl-1 and Fl-2 was determined with by flowcytometry. The ratio (Fl-2/Fl-1) represents the coupling efficiency (Δψ_m_) of the mitochondria as expressed as a percentage of the control (2 hour incubation without inhibitor). The average initial value for neutrophils was 3.32±0.85 (SD) and for PBMC 3.41±0.75 (SD). The F_0_ inhibitor oligomycin (A) and the F_1_ inhibitor aurovertin B (B) had a protective effect in all cells, demonstrating that reversal of complex V does not maintain Δψ_m_ in neutrophils. The complex III inhibitor antimycin A (C) completely reduced Δψ_m_ in all cells, while the complex-I inhibitor rotenone (D) did not affect neutrophils cultured with glucose. Data represent the means (±SEM) of five independent experiments performed in duplicate.

Because complex V displayed normal activity in neutrophils and represents the only remaining active complex capable of maintaining Δψ_m_ in neutrophil mitochondria, complex V was inhibited with increasing concentrations of either the F_0_-ATPase inhibitor oligomycin or the F_1_-ATPase inhibitor aurovertin B. No decrease in Δψ_m_ was observed in either neutrophils or PBMC (Figures 3A and 3B). However, oligomycin did cause a collapse of Δψ_m_ at concentrations above 1 µM in both cell types, with or without glucose. This is probably due to an aspecific effect of the inhibitor at high concentrations in intact cells, since even the PBMC, which have a normal respiratory chain, were affected. Generally, titration with the complex V inhibitors induced a rise in Δψ_m_, as expected in cells with respiring mitochondria. After all, complex V normally lowers Δψ_m_ by allowing protons to flow back into the mitochondrial matrix along with the gradient produced by the other complexes.

Inhibition of complex III by antimycin A annihilated the Δψ_m_ in both cell types, with neutrophils slightly more sensitive to low concentrations of this irreversible inhibitor than PBMC (Figure 3C). The higher sensitivity of neutrophils to this inhibitor can be explained by the fact that neutrophils contain relatively small amounts of complex III as compared to PBMC (Figure 2).This observation indicates that complex III, although showing little cytochrome *c*-reducing activity in neutrophils, still plays an important role in maintaining Δψ_m_ in these cells.

Inhibition of complex I by titration of rotenone also reduced the Δψ_m_ in both neutrophils and PBMC in the absence of glucose, although the inhibitory effect was larger in PBMC (Figure 3D). In the presence of glucose, increasing concentrations of rotenone had a slightly protective effect on Δψ_m_ in neutrophils, whereas in PBMC rotenone did reduce Δψ_m_, albeit not as pronounced as in the absence of glucose. These diminished effects are probably due to a combination of complex II activity compensating for the loss of electron flux from complex I and of ATP hydrolysis by complex V.

### Neutrophil mitochondria control lactate production but hardly contribute to ATP levels

**Figure 4:**
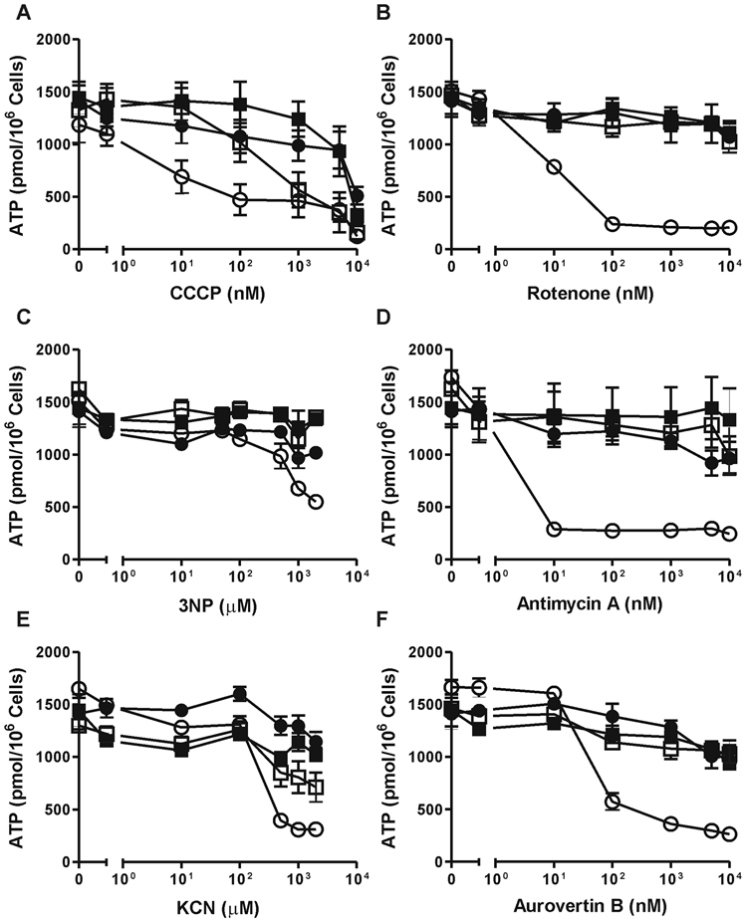
ATP levels in neutrophils and PBMC after treatment with respiratory chain inhibitors. A–F) Neutrophils (▪,□) and PBMC (•,○) were incubated with (▪, •) or without (□, ○) 5 mM glucose. After 2 hours of pre-incubation at 37°C, the cells were incubated for an additional 2 hours in the presence of various concentrations of the mitochondrial uncoupler CCCP (A), or inhibitors for the OXPHOS complexes I-V(F_1_), rotenone (B), 3-nitropropionate (3NP; C), antimycin A (D), KCN (E), or aurovertin B (F), repectively. ATP levels were determined with a luciferase-based assay. Data represent the means (±SEM) of three independent experiments performed in duplicate.

**Figure 5:**
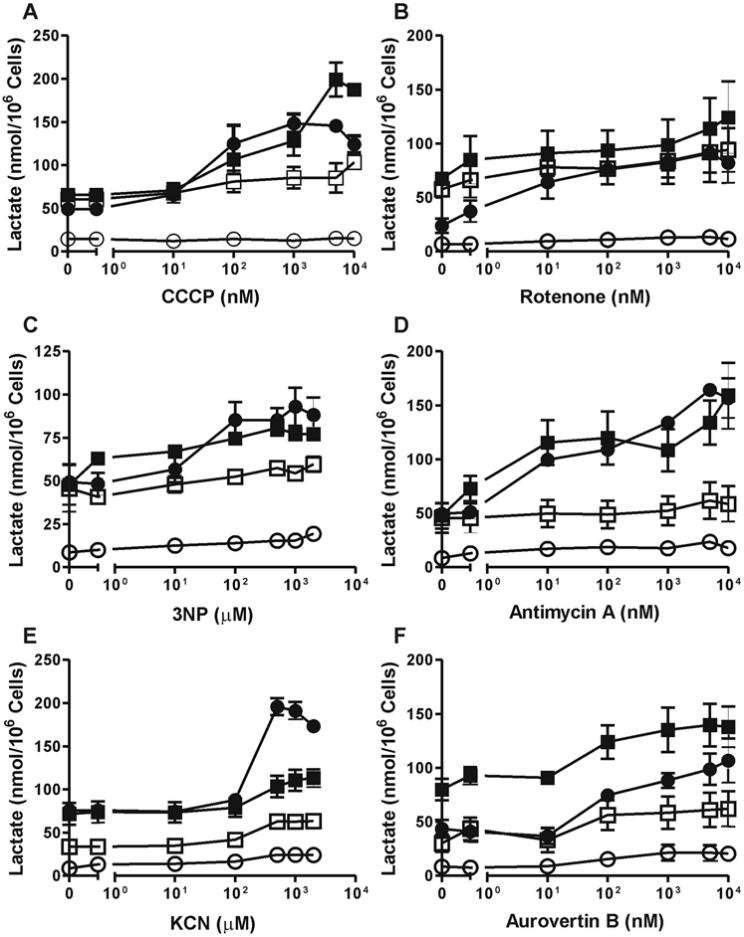
Lactate levels produced by neutrophils and PBMC after treatment with respiratory chain inhibitors. A–F) Neutrophils (▪,□) and PBMC (•,○) were incubated with (▪, •) or without (□, ○) 5 mM glucose. After 2 hours of pre-incubation at 37°C, the cells were incubated for an additional 2 hours in the presence of various concentrations of the mitochondrial uncoupler CCCP (A), or inhibitors for the OXPHOS complexes I-V(F_1_), rotenone (B), 3-nitropropionate (3NP; C), antimycin A (D), KCN (E), or aurovertin B (F), respectively. Lactate levels were determined with an enzymatic assay. Data represent the means (±SEM) of three independent experiments performed in duplicate.

To investigate the role of respiratory chain enzyme activity in maintaining the ATP levels of intact neutrophils and PBMC, cells were incubated with or without 5 mM glucose in the presence of increasing concentrations of specific OXPHOS inhibitors. After 2 hours of starvation, followed by a 2-hour incubation period with the inhibitor, the cells were lysed and ATP and lactate levels were determined in the neutralised lysates (Figures 4 and 5, respectively).

ATP levels in the control cells, i.e. PBMC cultured without glucose, proved particularly sensitive to the mitochondrial uncoupler carbonyl cyanide 3-chlorophenylhydrazone (CCCP; Figure 4A), to rotenone (Figure 4B), to antimycin A (Figure 4D) and to aurovertin B (Figure 4F). These compounds affected the ATP levels of the PBMC at a concentration of 100 nM or less. The complex II inhibitor 3-nitropropionate (3NP, Figure 4C), which was preferred over tenoyltrifluoroacetone (TTFA) in intact cells owing to the aspecific effects of the latter (data not shown), and the complex IV inhibitor KCN (Figure 4E) were only effective in reducing the ATP levels of the PBMC at concentrations of >100 µM. This is probably due to the hydrophilic nature of these inhibitors.

In neutrophils, the ATP levels remained largely unaffected by any of the inhibitors tested. Only CCCP (Figure 4A) caused a complete loss of ATP in neutrophils at a concentration of 10 µM. However, at this concentration the plasma membrane potential is also affected by this uncoupling agent (results not shown and [22]). This may lead to compensation by the Na/K-exchangers and rapid consumption of ATP. At 100 nM CCCP, the ATP levels in neutrophils incubated without glucose were reduced by about 30%, whereas this concentration did not affect the plasma membrane potential (results not shown). This suggests only a partial requirement for Δψ_m_ in maintaining ATP levels in neutrophils, since Δψ_m_ is completely lost at this concentration of CCCP (not shown). Similarly, the inhibitors KCN (Figure 4E) and aurovertin B (Figure 4F) decreased ATP levels in neutrophils by about 30% at concentrations above 100 µM and 100 nM, respectively. In PBMC, these concentrations were much more effective, leading to a decrease in ATP to less than 10% of the normal levels. Apparently, the OXPHOS system only partly maintains ATP levels in starving neutrophils.

Cells incubated in the presence of glucose seemed almost impervious to inhibition of the respiratory chain, as indicated by ATP levels. However, lactate production in both cell types increased significantly in response to increased levels of the inhibitors (Figure 5). Even in the absence of glucose (and inhibitors), neutrophils displayed about five-fold higher lactate production than PBMC, indicative of the ability of the former cells to utilize glycogen as an alternative energy source [3]. This may also explain why ATP levels in intact neutrophils were not as profoundly affected as in PBMC by mitochondrial inhibition in the absence of glucose (Figure 4).

In response to inhibition of complex I, II, III and V (F_1_), the increase in lactate production in the presence of glucose was similar for both neutrophils and PBMC, although the starting level of lactate produced by neutrophils cultured in the presence of glucose was generally higher (Figure 5). However, in response to the complex IV inhibitor KCN, PBMC produced significantly more lactate than did neutrophils, further indicating the strong dependency on the intact OXPHOS system of the former cells in contrast to the latter, which are adapted to low levels of complex IV by shuttling electrons from complex III directly to molecular oxygen, thereby generating superoxide [7].

### Neutrophils acquire their mitochondrial phenotype during differentiation

**Figure 6:**
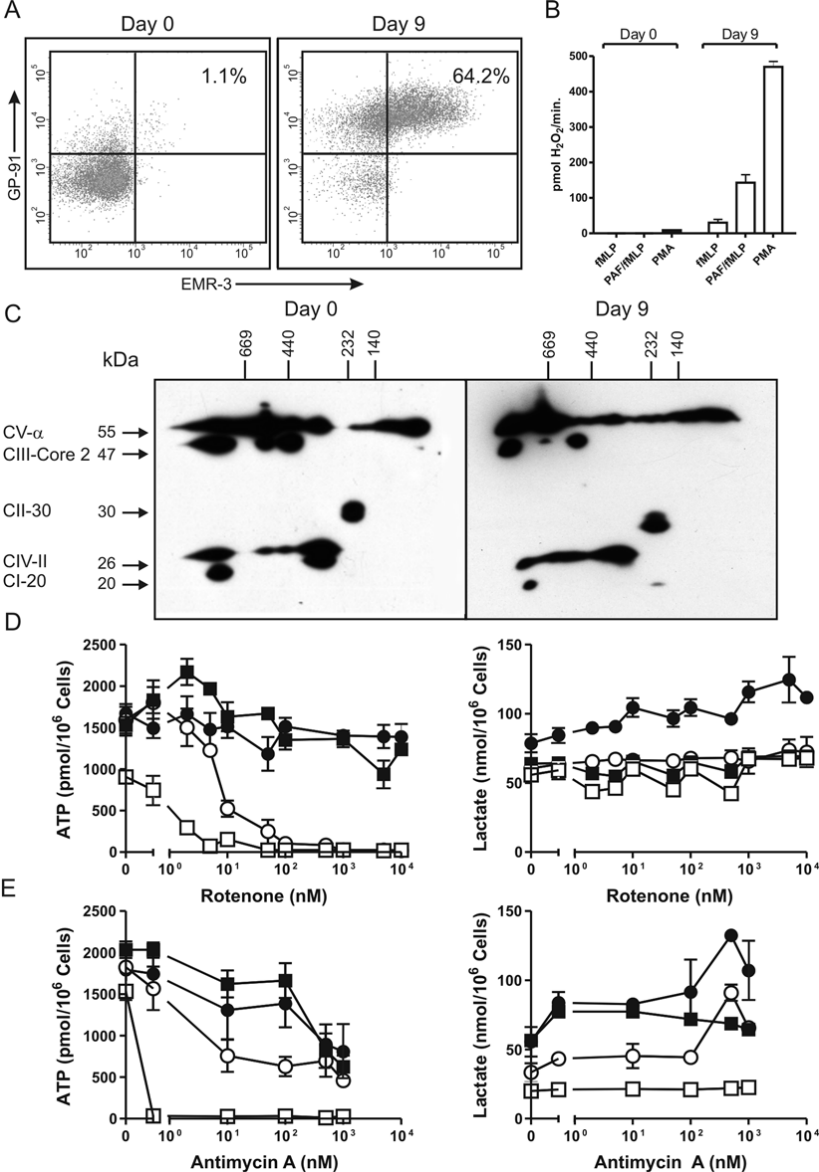
HL-60 cells loose supercomplex organisation and acquire neutrophil-like properties during differentiation. HL-60 cells were differentiated to neutrophil-like cells in 9 days. To demonstrate that HL-60 cells gain a neutrophil-like morphology and abilities during differentiation, the expression of the neutrophil markers gp91^PHOX^ and EMR3 was determined by flowcytometry (A) and their ability to produce a respiratory burst was determined by an Amplex Red assay (B). On day 9 of differentiation, approximately 60% of the cells stained positive for both neutrophil markers, while responding to all three stimuli (PMA, fMLP and the combination of PAF and fMLP) by producing a respiratory burst. Plots are representative of four independent experiments. C) Supercomplex organisation in HL-60 mitochondria before (Day 0) and after (Day 9) differentiation. Respiratory chain complexes were detected on Western blots of 2D-native/reduced SDS-PAGE gels with anti-complex I (20 kDa), complex II (30 kDa), complex III (Core 2, 47 kDa), complex IV (COX II, 26 kDa) and complex V (α, 55 kDa) subunit antibodies. Blots are representative of four independent experiments. During differentiation, HL-60 cells gain the metabolic properties of neutrophils (D and E). Undifferentiated (▪,□) and differentiated (•,○) HL-60 cells were incubated with (▪, •) or without (□, ○) 5 mM glucose. After 2 hours of pre-incubation at 37°C, the cells were incubated for an additional 2 hours in the presence of various concentrations of the complex-I inhibitor rotenone (D) or the complex-III inhibitor antimycin A (E) as indicated. Afterwards, ATP (left panels) and lactate (right panels) levels were determined. Data represent the means (±SEM) of three independent experiments performed in duplicate.

HL-60 cells can be differentiated to neutrophil-like cells in 7–9 days by stimulating the cells with 1.25% DMSO in the culture medium [6]. During differentiation, HL-60 cells gain neutrophil-like morphology and functions. Up to 70% of the cells become double positive for the neutrophil markers gp91^phox^ and EMR3 [23], respectively (Figure 6A) while the cells also gain the ability to produce a respiratory burst after stimulation with formyl-methionyl-leucyl-phenylalanine (fMLP), the combination of platelet activating factor (PAF) and fMLP or phorbol myristate acetate (PMA) (Figure 6B). Before differentiation (Day 0), the supercomplex organisation in the mitochondria of HL-60 cells is similar to the organisation found in PBMC (Figure 6C). After 9 days of differentiation, HL-60 cells have lost some of this organisation. In particular, complex I expression is significantly reduced in the large 1.5 MDa supercomplex. In the 370 kDa supercomplex, complex I is no longer detectable while a complex I spot appears around the same molecular weight as complex II (∼250 kDa). The expression of complex IV is also reduced during HL-60 differentiation. Especially the large 1.5 MDa supercomplex displays reduced levels of complex IV after differentiation. Similarly, expression of complex III is reduced in differentiated HL-60 cells, especially from the 600 kDa supercomplex.

While HL-60 cells loose supercomplex organisation, they gain resistance to the complex-I inhibitor rotenone (Figure 6D) and the complex-III inhibitor antimycin A (Figure 6E). In the presence of glucose, both differentiated and undifferentiated HL-60 cells are resistant to rotenone and antimycin A, but in the absence of glucose higher concentrations of these inhibitors is required to affect ATP levels in the differentiated cells than in the undifferentiated cells (Figures 6D and E). Differentiated HL-60 cells incubated in the presence of glucose respond to inhibitor titration by producing increasing amounts of lactate, just like neutrophils.

### Neutrophils can maintain Δψ_m_ by oxidation of glycerol-3-phosphate

Neutrophils have a relatively high rate of glycolysis, and this process seems thus to provide all the energy needed for phagocytosis and the respiratory burst [3]. During this process, NAD^+^ is reduced, requiring NADH to be reoxidised to keep the glycolytic pathway running. This can be done by either reducing pyruvate to form lactate, which normally occurs under anaerobic circumstances, or by reducing dihydroxyacetone phosphate (DHAP) to form glycerol-3-phosphate (G3P). G3P can diffuse into the mitochondria, where it is reoxidised to DHAP by mitochondrial flavin adenine dinucleotide (FAD)-dependent glycerol phosphate dehydrogenase (mGPD) on the outer surface of the inner mitochondrial membrane. Electrons from G3P are subsequently transferred to complex III of the respiratory chain via ubiquinol.

**Figure 7:**
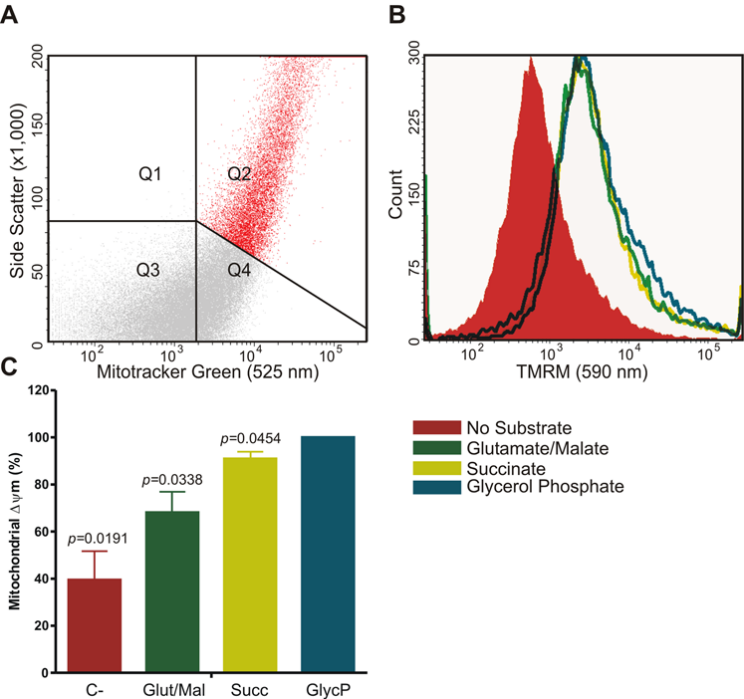
Membrane potential of isolated neutrophil mitochondria. Isolated neutrophil mitochondria were analysed by flow cytometry after staining with Mitotracker Green, to differentiate intact mitochondria from cellular debris, and TMRM to determine Δψ_m_. A) Scatter plot displaying intact mitochondria, defined as Mitotracker-Green-positive events (FITC channel) with high side scatter in Q2. B) Intact mitochondria gained TMRM staining (Δψ_m_, PE channel) after incubation with mitochondrial substrates. The complex-I substrates glutamate/malate (green) and the complex-II substrate succinate (yellow) induced a lower Δψ_m_ than glycerol phosphate (blue). Graphs are representative of three independent experiments. C) Graphic representation of the pooled experiments. Relative Δψ_m_ is expressed as a percentage of the mean fluorescence intensity (MFI) in the PE channel of the mitochondria population (Q2 in Figure 7A). The MFI of glycerol phosphate (GlycP) treated mitochondria was set at 100% while the MFIs of the untreated (C-), glutamate/malate (Glut/Mal) and succinate (Succ) treated cells were expressed as a percentage of this value. The average original value for C- was 1701±498 (SEM), for Glut/Mal 4442±2689 (SEM), for Succ 5481±3106 (SEM) and for GlycP 5838±3139 (SEM). Bars represent the means (±SEM) of three independent experiments.

In this respect, the response of both cell types to complex III inhibition was particularly striking, with a three-fold increase in lactate production (Figure 5D), as would be expected since the G3P shuttle delivers electrons directly to complex III. Isolated mitochondria from neutrophils can be energised by glycerol phosphate, as demonstrated by staining with tetramethylrhodamine-methylester (TMRM), proving that the mGDP is present and functional (Figure 7). The Δψ_m_ generated with glycerol phosphate as a substrate was significantly higher (P<0.05) than with the complex I substrates glutamate/malate, the complex II substrate succinate or in the absence of substrate (Figure 7C).

## Discussion

Even though neutrophils retain some activity of the respiratory chain complexes involved in OXPHOS, the present study shows that this activity is mostly required to maintain Δψ_m_. In PBMC and HL-60 cells, which have an active respiratory chain and do depend on OXPHOS activity to maintain their ATP levels, the respiratory chain is organised in supercomplexes. During differentiation of HL-60 cells to neutrophil-like cells, this supercomplex organisation is partly lost while the cells gain the metabolic properties of neutrophils. The inefficiency of neutrophil mitochondria to couple Δψ_m_ to OXPHOS can be explained by a decreased expression of the respiratory complexes I, III and IV, and the lack of their ability to assemble into supercomplexes.

Neutrophils possess a reduced copy number of mtDNA compared to HL-60 cells and PBMC, a defect that arises during differentiation [6]. Since most subunits of the respiratory chain complexes-and all subunits of complex II-are encoded in the nucleus, but several key proteins are still encoded by mitochondrial DNA (mtDNA), a decreased expression of all OXPHOS complexes except for complex II is not difficult to envision, but the consequences are far reaching. It has been shown in several human disorders that a defect in mtDNA leads to a different composition of the respiratory chain complexes and supercomplexes in skeletal muscle [24], [25]. However, assembly and stability of complex I not only depend on the correct information in the nuclear and mitochondrial genome, but also on whether respirasomes can be formed with complex III [26], [27]. While fully assembled complex I has a molecular mass of about 900 kDa, seven distinct complex I assembly intermediates ranging from 200 to 650 kDa have been identified in patients with complex I deficiency [28]. In addition, critical amounts of complexes III and IV are required for respiratory supercomplexes to assemble and to provide mitochondrial functional complementation [25]. Moreover, the expression of two forms of a supercomplex consisting of complex III and IV, namely III_2_IV_2_ and III_2_IV_1_, and free complex III depends on the growth conditions of the cells [18]. In neutrophils, only free complex III was demonstrable. The importance of the remaining complex III is stressed by our finding that lactate production, while being already high from the beginning, still increases when this complex is inhibited, and the fact that Δψ_m_ in neutrophils is lost upon incubation with antimycin A. Complex IV showed only residual activity in neutrophils, although very high concentrations of the complex IV inhibitor KCN (≥500 µM) did affect the ATP levels in starved neutrophils. Complex IV did not form supercomplexes with other respiratory chain complexes. As a consequence of the minor activity of complex IV, the electrons that flow from the proximal end of the respiratory chain will remain in the Q-cycle of complex III unless they are transferred on a one-to-one basis to molecular oxygen. While we have shown that the residual components of respiratory chain are not important for the production of ATP, maintenance of the Δψ_m_ in neutrophils will thus be at the cost of a relatively high mitochondrial superoxide production [7]. The majority of the required energy is provided by the G3P shuttle via mGPD as indicated by our finding that inhibition of complex III, but not so much of complex I or II, leads to a significant increase in lactate production in neutrophils. This mechanism also explains the fact that rotenone only affects Δψ_m_ in neutrophils in the absence of glucose. Under such starvation conditions, pyruvate will not be converted to lactate, but its full energy content will be utilized to produce NADH and FADH_2_ by which the remainder of complex I can play a (modest) role in maintaining Δψ_m_.

It is quite striking that the PBMC displayed such remarkable sensitivity for the mitochondrial inhibitors in the absence of glucose, as far as their ATP levels are concerned. Apparently, the absence of extracellular glucose is a strong stimulus for PBMC to utilize their mitochondria for their energy production, while aerobic glycolysis seems to be inhibited under these conditions. Starving cells may switch to fatty acid metabolism, which is completely dependent on mitochondria. As a consequence, the cells become more sensitive to mitochondrial inhibition, as shown in figures 4 and 5. The ATP levels in undifferentiated HL-60 cells are affected by starvation alone, as shown in figure 6D and E. Apparently, these cells are not selected for the ability to use substrates other than glucose very efficiently. On the other hand, starvation alone is not sufficient to affect the ATP levels in the differentiated cells. This might indicate that differentiation stimulates the cells to store glucose in the form of glycogen.

Complex I was not detectable on the BN/SDS-PAGE of neutrophil mitochondria (Figure 2B), but only on a (denaturating) Western blot (Figure 1C). Its protein expression was estimated to be only about 1% of that present in HL-60 cells and about 6% of that in PBMC. In the latter two cell types, complex I apparently assembles into two supercomplex forms, of about 1.5 MDa with complex III and IV, and of about 370 kDa with complex IV only (Figures 2A and 6C). Since a fully assembled complex I has an apparent molecular mass of about 900 kDa, the smaller supercomplex in HL-60 and PBMC must, therefore, contain an assembly intermediate of complex I as found in complex-I-deficient patients [28]. The subcomplex II is a likely candidate, since it contains the subunit NDUFS7 of 20 kDa to which the reference antibody was directed (Figures 2A and 6C) and has the proper apparent molecular mass of 230 kDa. We seem to detect this assembly intermediate around 230 kDa in differentiated HL-60 cells (Figure 6C), where it is not associated with either complex III or complex IV.

In neutrophils, the activity of complex V was comparable to that in PBMC and HL-60 cells, but its relative protein expression is 50% less than in PBMC and about 3-fold less than in the myeloid precursor HL-60. Besides the monomeric (∼600 kDa) and dimeric (∼1.5 MDa) forms, various subcomplexes of complex V were detected (Figure 2) including the assembly intermediates of about 370 and 470 kDa in the three cell types under study, and even smaller complexes still containing subunit α in PBMC and HL-60 cells. Such subcomplexes of complex V are indicative for mitochondrial biosynthesis disorders [13]. In that respect, neutrophils in particular resemble the mitochondrial DNA depletion syndrome [24], in accordance with the low mtDNA copy number found earlier by us [6]. In such patients, subcomplexes of complex V lack the F_0_-subunit [13]. This generally leads to less efficient coupling of respiration to ADP phosphorylation [13], [24]. Recent evidence indicates that complex V has a strong influence on the shape and architecture of the mitochondria due to its size and the tendency of F_0_ to form dimers in the mitochondrial membrane [29], [30]. A decreased expression of such dimers in neutrophil mitochondria might explain the peculiar tubular shape of these mitochondria [6], [7], and their inability to couple Δψ_m_ to ATP synthesis.

In summary, we show for the first time that the Δψ_m_ in neutrophils is maintained by complex III of the respiratory chain in the absence of detectable complex IV activity and not by complex V, as has been suggested for eosinophils [31]. This complex III activity does not contribute significantly to the energy status of the cells, while the energy for maintaining Δψ_m_ is mainly derived from the G3P shuttle (Figure 7). Thus, the role of neutrophil mitochondria is not restricted to apoptosis, but is also important in the regulation of glycolysis. Neutrophil mitochondria do not contain respirasomes and, as a consequence, they do not couple Δψ_m_ to efficient respiration and ATP synthesis. This particular phenotype probably arises during maturation from myeloid precursor cells, since we found that the mitochondria from HL-60 cells do contain respirasomes, while they loose some supercomplex organisation during differentiation to a neutrophil-like phenotype (Figure 6). These findings demonstrate the diversity of mitochondrial organisation within the human organism, and the implications of this organisation for cellular functions. In addition, they provide insight in the nature and consequences of mitochondrial biosynthesis disorders as well as indicating neutrophils as a model system for these disorders. The implications of these findings for neutrophil survival warrant further investigation.

## Materials and Methods

### Primary cell isolation and culture

Neutrophils and PBMC were isolated from heparinised blood of healthy volunteers, after informed consent had been obtained, by density gradient centrifugation over isotonic Percoll (Pharmacia, Uppsala, Sweden) and erythrocyte lysis [32]. PBMC were defined as the cells in the interface above the Percoll layer. Cells were cultured in Hepes-buffered saline solution (HBSS, 132 mM NaCl, 20 mM Hepes, 6 mM KCl, 1 mM MgSO_4_, 1.2 mM K_2_HPO_4_, 1 mM CaCl_2_, pH 7.4; chemicals from Merck) at a concentration of 10^7^ cells/mL in polypropylene round-bottom tubes of 14 mL (Falcon; BD; Franklin Lakes, NJ, USA). The medium was supplemented with 5 mM glucose where indicated. The incubation volume was never larger than 2.5 mL. All incubations were performed in a shaking water bath at 37°C.

### Culture and differentiation of HL-60 cells

HL-60 cells were cultured in Iscove's modified Dulbecco's medium (BioWhittaker, Brussels, Belgium) supplemented with 10% heat-inactivated foetal calf serum (Gibco BRL, Paisley, United Kingdom), 100 IU/mL penicillin (Gibco BRL), 100 µg/mL streptomycin (Gibco BRL), 300 µg/mL L-glutamine (Gibco BRL) and 50 µM β-mercaptoethanol (Gibco BRL), at 37°C, 10% CO_2_, until use.

Cells were differentiated in the same medium, supplemented with 1.25% dimethyl sulfoxide (DMSO; J.T. Baker BV, Deventer, The Netherlands) at a concentration of 0.5*10^6^ cells/mL. Every other day, the cells were counted and diluted to 0.5*10^6^ cells/mL in IMDM+1.25% DMSO. On day 0 and 9 of the differentiation the cells were harvested. To determine the differentiation state, cells were tested for the expression of the neutrophil markers EMR3 (clone 3D7, biotinylated F(ab')_2_ fragments, a kind gift from Dr. J. Hamann at the Academic Medical Centre in Amsterdam, The Netherlands [23]) and gp91^phox^ (clone 7D5, Sanquin, Amsterdam, The Netherlands). Labelling was performed in HBSS on ice for 20 minutes with the primary antibodies, after which the cells were washed and incubated with APC-labelled streptavidin (BD) to detect EMR3 and Alex Fluor-488-labelled goat-anti-mouse-IgG (Molecular Probes, Eugene, OR, USA) to detect gp91^phox^. Fluorescence was detected with an LSRII flowcytometer (BD). Quadrant settings were based on the background staining of non-differentiated cells. In addition, morphology of the cells was routinely checked by microscopy of cytospins stained with May-Grünwald-Giemsa stain.

### NADPH-oxidase Activity

Activity of the NADPH-oxidase in differentiated HL-60 cells was determined with an Amplex Red assay (Molecular Probes) after stimulation with 1 µM fMLP, the combination of 1 µM fMLP and 1 µM PAF, added simultaneously, or 100 ng/mL PMA, as described [33].

### Isolation of Mitochondria and Oxidative Phosphorylation Enzyme Activities

Cells were resuspended at a concentration of 5 mg/mL protein in mito buffer (0.2 mM EDTA, 0.25 mM sucrose, 10 mM Tris.HCl, pH 7.8) supplemented with a protease inhibitor mixture (PIM) of two Complete tablets per 50 mL (Roche Diagnostics, Almere, The Netherlands), 1 mM 4-(2-aminoethyl)-benzenesulfonyl-fluoride hydrochloride (Pefabloc SC, Roche) and 2 mM diisopropyl fluorophosphate (DFP; Fluka Chemica, Steinheim, Switzerland), freeze/thawed in liquid nitrogen and, after the addition of 10 mM triethanolamine and 0.1 mg/mL digitonin, incubated for 10 min on ice, homogenised in a pre-cooled glass-Teflon Potter-Elvehjem homogenizer, and centrifuged at 1000x*g* for 10 min at 4°C. The supernatant was saved, the pellet resuspended in the same volume of mito buffer supplemented with 0.1 mg/mL digitonin, homogenised and centrifuged once again. The combined supernatants were centrifuged at 12000x*g* for 15 min at 4°C, and the mitochondria-rich pellet was resuspended in mito buffer at the desired protein concentration.

OXPHOS complexes were determined according to Birch-Machin & Turnbull [34] with some essential modifications. The spectrophotometric methods were scaled down by the use of sub microcell quartz cuvettes (Hellma GmbH, Müllheim, Germany) of 100 µL, by which the concentration of cells could be increased without requiring large amounts of blood. Complex I activity was determined as the rotenone-sensitive oxidation of NADH with ubiquinone_1_ as electron acceptor (100 µM final concentration) at 340 nm, with 380 nm as the reference wavelength [35]. To assess complex II activity the extinction difference between 600 and 520 nm was used to follow the ubiquinone_2_-coupled TTFA-sensitive reduction of 2,6-dichlorophenolindophenol, with succinate as the substrate. Complex III was measured as the change in extinction difference between 550 and 540 nm for the antimycin-A-sensitive reduction of cytochrome *c* by ubiquinol_2_. Complex IV was determined by following the oxidation of reduced cytochrome *c* at 550 nm, with 540 nm as the reference wavelength in the presence of n-dodecyl-β-D-maltoside. Complex V was measured as oligomycin-sensitive Mg-ATPase activity immediately after brief sonication of the neutrophils according to Rustin *et al.* [36]. All inhibitors and substrates were obtained from Sigma (St. Louis, MO, USA).

The OXPHOS complex activities were expressed per mg protein as determined by the Bio-Rad DC protein assay (Bio-Rad Laboratories, Inc., Veenendaal, The Netherlands) with BSA as a standard, or normalized to the activity of citrate synthase determined according to Srere [37]. All assays were performed at least in duplicate.

### Blue-Native PAGE and Western-blotting

Mitochondria were incubated in lysis buffer (50 mM NaCl, 5 mM aminocaproic acid, 50 mM imidazole, pH 7.0) supplemented with PIM and digitonin (4 g/g mitochondrial protein) for 10 min on ice, and 200 µg of mitochondrial protein was further processed for first dimension BN-PAGE according to Schägger [38] on a 5–13% acrylamide gradient gel. Strips from the first dimension BN-PAGE were then excised, reduced for one hour at room temperature in 1% SDS containing 4 µL/mL tributylphosphine, and used for second dimension SDS-(12%)PAGE.

Western blotting was performed in a fully submerged Mini Trans-blot system (Bio Rad) using CAPS buffer (10 mM 3-[cyclohexylamino]-1-propane sulfonic acid, pH 11, 10% methanol) on PVDF membrane, after which the cornerstone subunit of each of the five OXPHOS complexes were visualized with the OXPHOS Western blotting kit (MS601; Mitosciences), biotinylated anti-mouse IgG (RPN1001, Amersham Biosciences), streptavidin-biotinylated horseradish peroxidase complex (RPN1051; Amersham) and SuperSignal West Femto (Pierce). Images were acquired using a calibrated densitometer (GS-800; Bio-Rad) and the PDQuest software package (version 6.2.1; Bio-Rad).

### Determination of Δψ_m_

To assess Δψ_m_ in neutrophils and PBMC, cells were incubated at 10^7^ cells/mL in HBSS with or without 5 mM glucose. After 2 hours of pre-incubation at 37°C the cells were incubated for an additional 2 hours in the presence of various concentrations of the indicated inhibitors in a shaking incubator. All inhibitors were obtained from Sigma and dissolved in dimethyl sulfoxide (DMSO). The total amount of DMSO added to the cells was 1% (v/v) for all incubations.

After incubation, the cells were stained by addition of 0.5 µM of the fluorescent dye JC-1 (all probes from Molecular Probes, Eugene, OR, USA) for an additional 15 minutes at 37°C. To determine a background value for Δψ_m_, cells were incubated with 1 µM CCCP (Calbiochem, La Jolla, CA, USA), which is sufficient to completely abrogate Δψ_m_. Red (Fl-2) and green (Fl-1) fluorescence was subsequently determined on a BD FACScan flowcytometer. The red-to-green ratio was determined by dividing the geometric mean fluorescence in Fl-2 by that in Fl-1. This value was corrected by subtracting the value obtained from the CCCP incubation and expressed as a percentage of the DMSO control. All assays were performed in duplicate.

### ATP and lactate assays

Incubation conditions were the same as mentioned above for Δψ_m_ determination. After incubation, the cells were lysed by the addition of 1.5% HClO_4_ for 10 minutes on ice. After centrifugation the supernatant was neutralized with an equimolar amount of K_2_CO_3_. Insoluble KClO_4_ was precipitated by quick centrifugation and the supernatant was stored at −80°C.

ATP levels were determined by adding 100 µL of the firefly luciferase based adenosine 5′-triphosphate (ATP) assay mix (Sigma) diluted 1∶10 in ATP-assay buffer (5 mM MgSO_4_, 100 µM EDTA, 100 µM NaN_3_, 4 mM DTT [DL-dithiothreitol, Sigma], in 25 mM TrisHCl, pH 7.8) to 10 µL of the supernatant of the neutralized lysate in a white 96-well plate (Corning Life Sciences, Corning, NY, USA). Samples were compared with an ATP standard (Sigma) treated in the same way as the samples. Luminescence intensity was determined immediately on a Spectra Fluor Plus spectrophotometer (Tecan, Zürich, Switzerland).

To determine lactate levels produced by the cells, 20 µL of the supernatant of the neutralized lysate was mixed with 100 µL of lactate assay solution (Trinity Biotech plc, Wicklow, Ireland). After 15 minutes of incubation at room temperature, absorbance was measured at 540 nm in the spectrophotometer.

### Analysis of Δψ_m_ in isolated mitochondria

The mitochondrial fraction from ∼150*10^6^ neutrophils was resuspended in 2 mL of mitochondria assay buffer (120 mM KCl, 5 mM KH_2_PO_4_, 1 mM EDTA, 1 mM MgCl_2_, 3 mM Hepes, pH 7.8, 1% (v/v) human serum albumin; Sanquin, Amsterdam, The Netherlands) and divided in 100-µL portions that were either left unsupplemented or supplemented with either 10 mM *sn*-glycerol 3-phosphate, the combination of 10 mM glutamate and 2 mM malate, or 10 mM succinate.

After 1 hour of incubation at 37°C, the mitochondria were stained for an additional 15 minutes with 1 µM Mitotracker Green, to differentiate intact mitochondria from cellular debris, and 100 nM TMRM to determine Δψ_m_. Samples were analysed on a LSRII flow cytometer (BD) with FACSDiva software. To detect small particles, forward scatter (FSC) was set to 600 and side scatter (SSC) to 550. Intact mitochondria were defined as having high side scatter (SSC) and high Mitotracker staining (FITC channel). Compensation between PE and FITC was set at 15%. At least 10,000 positive events were collected for each sample. All assays were performed in duplicate.

### Statistical analysis and image processing

Graphs were drawn and statistical analysis was performed with Prism 4.03 (GraphPad Software). The results are presented as the mean±s.e.m. or s.d., as indicated. Data were evaluated by paired, one-tailed student's *t*-test, where indicated. The criterion for significance was P<0.05 for all comparisons.

Images were processed in Adobe Photoshop CS (Adobe Systems Inc.) and CorelDRAW 11 (Corel Corporation).
